# Increasing nutrition knowledge and culinary skills in interprofessional healthcare students: an active learning pilot study

**DOI:** 10.1186/s12909-025-07247-y

**Published:** 2025-05-26

**Authors:** Tracy Noerper, Anne Lowery, Geoffrey Wright, Abigail Burka

**Affiliations:** College of Health Sciences, Lipscomb University, One University Park Drive, Nashville, TN 37204 USA

**Keywords:** Nutrition, Cooking, Food, Curriculum, Interprofessional education, Dietetics

## Abstract

**Background:**

Hands-on culinary training can equip healthcare students to educate patients on a healthy diet. Research suggests there is improved self-confidence in patient counseling interactions along with a deeper understanding on the role of an interprofessional healthcare team during hands-on culinary medicine courses. There is a paucity of evidence describing the outcomes that culinary training offers for interprofessional healthcare students in educating individuals who are vulnerable to poverty. The researchers suggested that an active learning, educational program would boost participants’ culinary skills, increase nutrition knowledge, and improve attitudes and confidence in counseling patients with limited means on a healthy diet.

**Methods:**

The study included a 4-week active learning, educational pilot where nutrition faculty and graduate-level dietetic interns taught nutrition and culinary skills to nursing, physician assistant, and pharmacy students. Each of the four, 2-hour educational sessions focused on cooking skills integrated within nutrition education on topics such as general nutrition, obesity, diabetes, and cardiovascular disease. Recipe selections were based on ingredient cost of $5.00 per person or less, readily accessible ingredients, and basic food preparation skills. Pre-, post-, and 2-month post-intervention survey data were collected and analyzed. Paired sample *t*-tests were computed to assess for changes between each of the study periods (*p* *≤* 0.05).

**Results:**

Data analysis indicated that participants (*N* = 14) had a high level of basic culinary skill knowledge at baseline with a mean score of 8.07 (*SD* = 0.78) out of 10 when compared to nutrition knowledge (*M* = 7.00, *SD* = 1.04). Statistically significant improvements (*p* *≤* 0.05) were found in pre- to post-assessment participant attitudes toward their counseling abilities in culinary knowledge [95% CI, 1.27 to 4.73; *p* = 0.002] and culinary techniques/skills [95% CI, 1.25 to 4.75; *p* = 0.002]. Post- to 2-month post-intervention analysis showed no statistically significant increased mean scores in attitudes, behaviors or counseling characteristics.

**Conclusions:**

Interprofessional healthcare students may benefit from education that supports improving nutrition knowledge as well as attitudes surrounding their counseling abilities and confidence on a healthy diet, especially with clients who have lower incomes. Future research of hands-on culinary education should aim to balance culinary skills education with improving the nutrition knowledge of interprofessional healthcare students.

**Supplementary Information:**

The online version contains supplementary material available at 10.1186/s12909-025-07247-y.

## Background

The healthcare burden of chronic disease has been documented in the United States (US) for nearly 20 years [1]. Global estimates indicate that 1 in 3 adults “suffer from multiple chronic conditions” with healthcare expenditures increasing with each additional chronic condition [2]. Chronic diseases such as diabetes, hypertension, coronary heart disease, and asthma are multifactorial in cause and related in part to a rise in chronic disease risk factors like obesity and an aging population [3].

Lower income populations have specifically faced the pressures of added chronic disease burden [1, 4]. For example, food insecure young adults have been found to have greater odds of self-reported poor health (e.g. diabetes, hypertension, overweight, obstructive airway disease) compared with young adults who were food secure [4]. Additionally, individuals with food insecurity have been found to incur higher healthcare costs and had poorer health compared to those who were food secure [5]. The term “food insecure” indicates that a household was uncertain of having, or unable to acquire, enough food to meet the needs of all their members because they had insufficient money or lacked other resources for food [6]. According to figures from the United States Department of Agriculture (USDA), 12.8% (17.0 million) of US households were food insecure in 2022, which was statistically significantly higher than the 10.2% (13.5 million) in 2021 [6, 7].

To combat food insecurity, the Supplemental Nutrition Assistance Program (SNAP), a federal nutrition program, is designed to provide supplemental funds to households that meet certain income requirements. In 2020, SNAP served an average of 41.5 million people monthly, approximately 12.5% of Americans [8]. A majority of SNAP participants (88%) have reported they face a variety of challenges in eating a healthy diet with 61% indicating that the cost of healthy food was a barrier in consuming a healthy diet [9]. SNAP participants indicate a limited knowledge about healthy foods and insufficient cooking skills as obstacles to maintaining a healthy diet. However, SNAP-eligible adults have expressed interest in nutrition education, particularly when it includes a hands-on cooking component [10].

Health professionals play a vital role in educating clients on healthy lifestyle habits, including those relating to nutrition and diet, yet training curricula for health professionals often lack a focus on nutrition-related competencies [11]. Previous research on the role of culinary courses for allied health professions has shown positive impacts on behaviors and knowledge among college-age students. Warmin and colleagues found that a chef and dietitian-guided cooking program was effective in increasing self-efficacy among college students and improving healthy eating behaviors [12]. Additionally, improved self-confidence in patient counseling interactions along with an understanding of the role of the interprofessional team has been shown effective in hands-on culinary medicine courses [13]. Nutrition education based on hands-on experiential learning in a kitchen setting has been shown as a successful strategy for “increasing counseling confidence” and “superior for acquiring and mastering new skills” since it allows the learner to “immediately apply new knowledge with peer support” and faculty or expert guidance [13]. Additionally, culinary skills, such as knife skills and cooking methods (e.g. boiling, roasting, etc.), have been successfully woven into culinary courses as a way to increase a student’s confidence and improve cooking behaviors [13, 14].

### The role of interprofessional education in culinary education

The inadequacy of nutrition education in medical and other health professions training has been well-described [15, 16, 17, 18]. Nurses, physician assistants, and pharmacists all report a desire to receive more nutrition education to better care for patients [19]. Culinary medicine is an evidence-based field that blends the art of food and cooking with the science of medicine [20]. Similarly, culinary nutrition is the application of nutrition principles combined with food science knowledge and displayed through mastery of culinary skills [21]. Hands-on culinary nutrition programs, that include food preparation, are an increasingly popular way to equip healthcare professionals with practical skills and knowledge to educate patients on a healthy diet. These workshops or programs are often led by professional chefs or registered dietitians and have traditionally involved participants from various health professions [22]. Although some interprofessional culinary education events have been previously described, much of the published literature has focused on the effect of culinary nutrition education on physicians and medical students with a lack of spotlight on other allied health professionals [22, 23, 24].

Importantly, access to affordable and high-quality nutrition is a social determinant of health which contributes to global chronic disease burden and premature deaths. Health professionals have the potential to benefit from increased knowledge of nutrition principles and culinary skills [25]. Culinary nutrition education lends itself to the interprofessional education model, where students learn about, from and with each other to enable effective collaboration and advance health outcomes [26]. Previous participants in interprofessional culinary nutrition education cite learning about nutrition topics, hands-on cooking, interprofessional networking, and socialization as reasons for attendance [27].

Hands-on culinary experiences have created the opportunity for interprofessional education (IPE) students to engage in teamwork, problem-focused learning, cooperation, effective communication, and the opportunity to develop a better understanding of others’ scopes of practice [27, 28]. Culinary nutrition courses utilizing a multi-discipline IPE approach generate an environment for students “to learn from, with and about other healthcare disciplines” in addition to gaining the culinary skills and knowledge provided during the experience [29]. Student feedback on culinary projects indicates that they gain not only the value of teamwork but also the perspectives of a variety of healthcare disciples [27, 29]. Understanding the roles and training of various healthcare providers is an important benefit of culinary activities [27, 30].

### Aims of the study

To explore the impact that a hands-on culinary nutrition program could have on IPE healthcare students, especially focusing on affordable recipes, a research pilot study was conceptualized among IPE university faculty representing the areas of dietetics, pharmacy, and physician assistant. The study aimed to: (1) evaluate the attitudes of various health professional students in working with clients on achieving healthy dietary behaviors, (2) increase confidence in effective counseling skills of health professional students through participation in a culinary nutrition experience, and (3) increase health professional student’s culinary skills and nutrition knowledge. In addition, the researchers desired to explore the current food preparation and dining behaviors of health professional students and determine if a hands-on culinary nutrition program had any impact on those behaviors.

## Methods

### Study design and participants

The study design was a quasi-experimental pilot study with a two-month follow-up period. The study was approved by the University’s Institutional Review Board (IRB) before study initiation. Participants were recruited from students enrolled in health sciences interprofessional education (IPE) at a university. An initial recruitment email and follow-up email were distributed by 3 University faculty members to all possible participants for 5 weeks before the study initiation. The recruitment email included an invitation to review the study parameters and consent documents. Inclusion criteria were that participants were current interprofessional education (IPE) students, 18 years of age or older, and provided electronic consent. Three hundred eighty-one IPE students were eligible for participation in the study from the areas of pharmacy, physician assistant, and nursing. Students who were enrolled in those areas, interested in the study, provided consent to participate and could attend the educational sessions in person were eligible to participate.

The culinary class experiences and curriculum were based on the social cognitive theory (SCT) and its methods for increasing self-efficacy. Within the SCT framework there are opportunities for social modeling, verbal persuasion, observational learning, reinforcement, and creating opportunities for small steps to achieve positive outcomes which have been successfully used to impact nutrition and health-related behaviors [31, 32, 33]. For example, combining nutrition education with live cooking demonstrations, using the SCT framework, was found effective in boosting diet quality by encouraging the consumption of homemade meals leading to improved knowledge and skills, and ultimately an increased intake of fruits, vegetables, and whole grains [31, 32, 33].

### Survey development

The data collection tool (i.e. survey) was adapted slightly from a previously published culinary intervention by Wood and colleagues, including the addition of an expanded nutrition knowledge section (10 questions) based on recommendations from that pilot [14, 34]. Subject matter experts (SMEs) from the areas of nutrition, physician assistant, pharmacy, and exercise science were recruited to review the survey tool for face and content validity. Validity results indicated that 100% of subject matter experts (*N* = 8) agreed with the face validity of the survey. The Content Validity Ratio (CVR) calculation for the survey was 0.67 which did not meet the 0.99 minimum CVR threshold required to establish content validity of the survey [35]. Minor survey edits were made based on SME feedback before distribution. See the supplemental section for the survey.

The distributed survey included 5 demographic questions followed by sections covering attitudes (6 questions), behavior (7 questions), and counseling patients on healthy lifestyles (6 questions) where participants rated their agreement with statements in these areas on a scale of zero (do not agree at all) to 10 (completely agree) or the number of times the activity occurred per week (0 to 10+) depending upon the question. Scoring of the attitudes and counseling survey sections included summing the reported scores and then calculating the means for each question, where a higher mean score indicated higher or complete agreement and a lower mean score indicated a lower or zero (0) agreement. The behavior survey section scoring also included summing the reported scores and calculating the means for each question where a lower number (0) indicated fewer times per week the behavior was reported while a higher mean score indicated a more frequent number of times the behavior occurred during a week (10+). The last 2 survey sections were multiple choice or matching questions covering culinary knowledge and skills (10 questions) and nutrition knowledge (10 questions) where participants selected what they believed to be the correct answer. Scoring of the culinary knowledge and skills section included assigning 1 point for each correct answer (e.g. 9 correct questions out of 10 possible questions equaled a score of 9) for a maximum score of 10. Question 8 contained 4 answer options (0.25 points for each correct answer) totaling 1 full point. Similarly, scoring the nutrition knowledge questions included assigning 1 point for each correct answer (e.g. 8 correct questions out of 10 possible questions would be a score of 8) for a maximum score of 10. Each question in the culinary knowledge and skills as well as the nutrition knowledge only had 1 correct answer.

The study included 44 survey questions, with an estimated completion time of approximately 15 min, as stated in the participant consent document. The same survey was distributed at each time frame (including demographic questions) in the study to assess for differences and continuity of responses. Survey responses were tracked via the student’s email address (part of the demographics section) in order to match pre-, post-, and 2-month follow-up data accordingly. Appropriate privacy and confidentiality measures of any identifiable data were accounted for and included in the IRB approval process.

### Culinary intervention

Participants attended a consecutive, four-week culinary class consisting of 2 hours per session. The no-cost class sessions were held on a university campus once per week for consecutive weeks in January and February of 2022 in the nutrition department’s food lab. The sessions were conducted from 5 to 7 pm to coincide with a typical evening meal preparation and consumption timeframe. Sixteen graduate-level, dietetic interns developed the weekly educational curriculum along with the associated recipes. Curriculum development and recipe testing by the same graduate interns were overseen by expert faculty in nutrition, food science, and food systems content. Recipe development included costing of the ingredients to meet a $5 per person per serving parameter for each meal. Additionally, recipes went through a testing phase with minor adjustments to ingredients and procedures based on peer and faculty feedback during the testing. Each of the four-week culinary sessions focused on specific nutrition topics and culinary skills (Table 1). Culinary sessions were comprised of 15 to 20 min of education on the week’s nutrition topic, 15 min of culinary skills demonstration (e.g. skills needed to prepare the recipe, viewing of the final recipe, etc.), and the remainder of the time the participants, working in groups of two, prepared the week’s recipe. After the recipe was prepared the participants sampled the prepared food items. The total food costs of the course were approximately $421 (in 2022), including the weekly demonstration recipe. The food and supplies for the classes were paid for through nutrition department funds. There was no charge for students to attend the classes.

**Table 1:** The weekly structure of the hands-on culinary nutrition classes

		Weekly Session		
Topics	Week 1	Week 2	Week 3	Week 4
Knife Skills	Cutting, Dicing	Peeling, Chopping	Coring, Slicing	Mincing
Cooking Skills	Boiling, Broiling	Sautéing, Simmering	Browning, Roasting	Steaming, Baking
Nutrition Topics	Basic Nutrition, Macronutrients, Recipe Structures, Food Safety	Obesity, Portion Size, Smart Substitutions	Diabetes, Carbohydrate Awareness, Healthy Starches	Cardiovascular Disease, Seasoning with Herbs and Spices
Recipes	Italian-style Turkey Meatballs, Pasta, Roasted Broccoli	Winter Harvest Salad with Whole Grain Bread	Fiesta Beef Burrito Bowls with Salsa Verde	Baked Pesto Salmon and Tomatoes with Steamed Broccoli

### Procedures & data analysis

Participants completed the identical electronic survey 3 times during the pilot study: pre-test (baseline), post-test (at the immediate conclusion of the 4-week intervention), and at a 2-month follow-up time. Students completed the electronic survey on their own electronic devices for increased privacy measures. Data was downloaded from the online platform and analyzed with IBM SPSS^®^ Statistics for Windows (Version 28.0. Armonk, NY: IBM Corp, 2021). Descriptive statistics, including mean scores and standard deviations, were computed for the 3 time periods. Paired sample *t*-test analyses were also performed and reported. Similar to other studies with multiple data collection points, the authors collected a baseline measurement (pre-test) before the culinary intervention, again immediately after the last culinary intervention (post-test), and at a 2-month post-test time point to assess if changes were sustained beyond the post-test period, providing a broad understanding of the intervention’s impact [14, 36]. For the analyses statistical significance was defined as *p* *≤* 0.05. There were no tests for any effect sizes due to the small participant size of the sample. Lastly, the statistical software used for analysis was performed using a pairwise deletion for any pair of data with a missing value and therefore did not include that data pair in the analysis. For example, since the number of responses was lowest (11) for the post-test evaluation, only those 11 responses with corollary values were included in the paired t-tests; therefore, eliminating any potential bias from missing values.

## Results

### Study participation

Fourteen student participants (*N* = 14) agreed to take part in the study (3.7% response rate) and indicated consent. Over the 4-week study, attendance ranged from 7 to 14 with an average of 9 participants at each session. Fourteen students completed the pre-test survey, 11 students for the post-test survey, and 13 students completed the 2-month follow-up survey. Participant demographics are described in Table 2.

**Table 2:** Demographics of the Hands-on Culinary Series Participants (*N*=14)

Characteristic	*N*	M
Age in Years (Range 20 - 42)	14	25.71
	*N*	*%*
Gender		
Male	5	36
Female	9	64
Race		
Black	2	14
Asian/Indian	3	21
Hispanic	3	21
White	6	43
Marital Status		
Married	3	21
Not Married	11	79
Participant Concentration		
Nursing	2	14
Physician Assistant (PA)	3	21
Pharmacy	9	65

Note: N = Number. M = Mean

### Statistical analysis

Descriptive statistics were computed for the 3 data collection periods (pre-, post-, and 2-month follow) and included each of the domain areas (i.e. attitudes, behavior, counseling, culinary knowledge and skills, and nutrition knowledge). Paired sample *t*-tests assessed for changes between each of the study periods.

The survey questions which covered attitudes focused on medical, nutrition, and culinary knowledge as well as the students’ overall motivation and skills related to their health. At baseline (Table 3), participants had the overall lowest average attitude scores in 2 areas: medical knowledge (*Mean (M)* = 6.07, *Standard Deviation* (*SD)* = 2.62) and culinary knowledge (*M* = 6.07, *SD* = 2.59) needed to practice a healthy lifestyle. The highest mean attitude baseline score (Table 3) was that participants perceived that culinary knowledge and skills could positively impact their health (*M* = 8.14, *SD* = 1.99).

**Table 3:** Pre-Test, Post-Test and 2-Month Follow-Up Mean Scores of Attitudes, Behaviors, Counseling and Knowledge Areas

	Pre-Test (*N*=14)	Post-Test (*N*=11)	2-Month Follow-Up (*N*=13)
	*M (SD)*	*M (SD)*	*M (SD)*
Attitudes - I have the…			
Medical knowledge	6.07 (2.62)	8.45 (1.21)	8.77 (0.93)
Nutrition knowledge	6.29 (2.67)	8.55 (1.21)	9.00 (1.16)
Culinary theory knowledge	6.07 (2.59)	8.64 (1.12)	8.46 (1.27)
Culinary techniques/skills	6.29 (2.87)	8.64 (1.21)	8.92 (0.95)
Motivation	7.64 (2.06)	8.91 (1.04)	8.85 (1.46)
Knowledge and skills for my health	8.14 (1.99)	9.09 (0.83)	8.69 (1.65)
Behaviors - Estimate the Number of Times Per Week			
Eat Meals from a Restaurant	3.50 (2.68)	2.91 (1.97)	3.92 (2.84)
Eat Pre-Prepared Meals (not from a restaurant)	2.29 (2.37)	2.36 (2.38)	2.15 (2.85)
Eat Breakfast at Home	5.29 (2.92)	6.55 (2.95)	6.15 (2.73)
Eat Lunch at Home	5.57 (2.34)	5.91 (2.77)	5.62 (2.57)
Eat Dinner at Home	5.50 (2.31)	4.82 (2.14)	5.00 (2.71)
Eat Leftovers from Meals Made at Home	6.29 (2.59)	4.45 (1.51)	5.23 (2.92)
Eat Pre-Prepared Meals Despite Preferring to eat at Home	3.50 (3.23)	3.27 (3.13)	2.85 (2.85)
Counseling Patients on Healthy Lifestyles			
I have the medical knowledge	6.71 (2.95)	8.64 (1.29)	8.69 (1.11)
I have the nutritional knowledge	5.64 (2.71)	8.64 (0.81)	8.69 (1.03)
I have the culinary knowledge	5.64 (7.34)	8.45 (0.82)	8.92 (1.04)
I am prepared to counsel patients	7.00 (1.66)	8.64 (0.81)	8.77 (1.24)
I am motivated to counsel patients	9.14 (1.10)	9.27 (0.91)	9.23 (0.83)
I am excited to counsel patients	9.36 (0.93)	9.36 (0.81)	9.23 (0.93)
Culinary Knowledge & Skills	8.07 (0.78)	8.80 (1.17)	8.42 (0.75)
Nutrition Knowledge	7.00 (1.04)	7.36 (1.75)	7.15 (1.73)

Note: Attitudes, Behavior and Counseling Patient sections were based on 10-point Likert scales. Culinary Knowledge & Nutrition Knowledge scores were out of 10 questions. N = Number. SD = Standard Deviation. M = Mean

Statistically significant differences in pre-test to post-test scores were found in all areas of attitudes (Table 4). Most notable were statistically significant changes in pre- and post-scores for attitudes towards participants medical knowledge [95% CI, 0.56 to 4.70; *p* = 0.01], nutrition knowledge [95% CI, 0.66 to 4.61; *p* = 0.01], culinary knowledge [95% CI, 1.27 to 4.73; *p* = 0.00], and culinary techniques/skills [95% CI, 1.25 to 4.75; *p* = 0.00]. Analysis of pre-test to 2-month follow-up of mean score changes continued with statistically significant results in all areas of participant attitudes except for attitudes surrounding knowledge and skills for participant health (Table 5). Post-test to 2-month analysis showed no statistically significant differences in mean scores in any area of attitudes (Table 6).

**Table 4:** Paired Sample T-test Results Comparing Pre- and Post-Test Change Scores on Attitudes, Behavior and Counseling (*N*=11)

Characteristic	M	SD	95% CI		t	df	(one-sided *p*)
			Lower	Upper			
**Attitudes**: I have the…							
Medical Knowledge	2.64	3.07	0.56	4.70	2.84	10	0.01*
Nutrition Knowledge	2.64	2.94	0.66	4.61	2.97	10	0.01*
Culinary Knowledge	3.00	2.57	1.27	4.73	3.87	10	0.00*
Culinary Techniques/Skills	3.00	2.61	1.25	4.75	3.83	10	0.00*
Motivation	1.45	1.97	0.13	2.78	2.45	10	0.02*
Knowledge And Skills For My Health	1.27	2.26	-0.26	2.81	1.85	10	0.05*
**Behaviors**: Number of Times per Week Eating…							
Meals from a Restaurant	-1.00	2.24	-2.50	0.50	-1.48	10	0.08
Pre-Prepared Meals	-0.45	3.08	-2.52	1.61	-0.49	10	0.08
Breakfast at Home	1.64	3.32	-0.60	3.87	1.63	10	0.07
Lunch at Home	0.64	1.5	-0.37	1.65	1.41	10	0.10
Dinner at Home	-0.27	3.9	-2.89	2.35	-0.23	10	0.41
Leftovers from Meals Made at Home	-1.55	2.3	-3.09	0.00	-2.23	10	0.03*
Pre-Prepared Meals Preferring to eat at Home	-1.00	3.95	-3.65	1.65	-0.84	10	0.21
**Counseling**: Patients on Healthy Lifestyles…							
Medical Knowledge	2.00	2.28	0.47	3.53	2.91	10	0.01*
Nutrition Knowledge	3.36	2.77	1.50	5.22	4.03	10	0.00*
Culinary Knowledge	3.18	3.09	1.10	5.26	3.41	10	0.00*
Prepared to Counsel Patients	1.82	1.66	0.70	2.94	3.63	10	0.00*
Motivated to Counsel Patients	0.36	1.29	-0.50	1.23	0.94	10	0.19
Excited to Counsel Patients	0.18	0.87	-0.41	0.77	0.69	10	0.25

Note: *significant one-tailed *p*<0.05; N = Number. M = Mean. SD = Standard Deviation. CI = Confidence Interval. df = Degrees of Freedom

**Table 5:** Paired Sample T-test Results Comparing Pre-Test to 2-Month Follow-Up Change Scores on Attitudes, Behavior and Counseling (*N*=13)

Characteristic	M	SD	95% CI		t	df	(one-sided *p*)
			Lower	Upper			
**Attitudes**: I have the…							
Medical Knowledge	2.23	1.79	1.15	3.31	4.50	12	0.00*
Nutrition Knowledge	2.23	2.35	0.81	3.65	3.42	12	0.00*
Culinary Knowledge	1.92	2.29	0.54	3.31	3.03	12	0.01*
Culinary Techniques/Skills	2.15	2.12	0.88	3.43	3.67	12	0.00*
Motivation	0.92	1.50	0.02	1.83	2.22	12	0.02*
Knowledge And Skills For My Health	0.69	2.39	-0.75	2.14	1.04	12	0.16
**Behaviors**: Number of Times per Week Eating…							
Meals from a Restaurant	0.54	2.57	-1.01	2.09	0.76	12	0.23
Pre-Prepared Meals	0.08	2.10	-1.19	1.35	0.13	12	0.45
Breakfast at Home	0.62	2.96	-1.17	2.40	0.75	12	0.23
Lunch at Home	-0.23	2.49	-1.74	1.27	0.33	12	0.37
Dinner at Home	-0.77	3.35	-2.79	1.25	0.83	12	0.21
Leftovers from Meals Made at Home	-1.39	3.33	-3.40	0.63	1.50	12	0.08
Pre-Prepared Meals Preferring to eat at Home	-0.31	4.13	-2.80	2.19	0.27	12	0.40
**Counseling**: Patients on Healthy Lifestyles…							
Medical Knowledge	1.46	1.33	0.66	2.27	3.96	12	0.00*
Nutrition Knowledge	2.62	2.18	-1.30	3.93	4.32	12	0.00*
Culinary Knowledge	2.85	1.99	1.64	4.05	5.15	12	0.00*
Prepared to Counsel Patients	1.69	1.75	0.64	2.75	3.49	12	0.00*
Motivated to Counsel Patients	0.00	0.71	-0.43	0.43	0.00	12	0.50
Excited to Counsel Patients	-0.23	0.93	-0.79	0.33	0.90	12	0.19

Note: *significant one-tailed *p*<0.05; N = Number. M = Mean. SD = Standard Deviation. CI = Confidence Interval. df = Degrees of Freedom

**Table 6:** Paired Sample T-test Results Comparing Post-Test to 2-Month Follow-Up Change Scores on Attitudes, Behavior and Counseling (*N*=10)

			95% CI				Significance
**Characteristic**	***M***	***SD***	**Lower**	**Upper**	***t***	**df**	(one-sided *p*)
**Attitudes**: I have the…							
Medical Knowledge	0.20	1.14	-0.61	1.01	0.56	9	0.30
Nutrition Knowledge	0.60	1.27	-0.31	1.51	1.50	9	0.08
Culinary Knowledge	-0.30	1.42	-1.31	0.71	-0.67	9	0.26
Culinary Techniques/Skills	0.10	1.37	-0.88	1.08	0.23	9	0.41
Motivation	0.00	1.05	-0.75	0.75	0.00	9	0.50
Knowledge And Skills For My Health	-0.30	1.83	-1.61	1.01	-0.52	9	0.31
**Behaviors**: Number of Times per Week Eating…							
Meals from a Restaurant	1.00	1.89	-0.35	2.35	1.68	9	0.06
Pre-Prepared Meals	0.00	3.86	-2.76	2.76	0.00	9	0.50
Breakfast at Home	-0.90	3.14	-3.15	1.35	-0.91	9	0.19
Lunch at Home	-1.00	2.49	-2.78	0.78	-1.27	9	0.12
Dinner at Home	-0.50	2.32	-2.16	1.16	-0.68	9	0.26
Leftovers from Meals Made at Home	0.50	2.55	-1.32	2.32	0.62	9	0.28
Pre-Prepared Meals Preferring to eat at Home	-0.10	4.95	-3.64	3.64	-0.06	9	0.48
**Counseling**: Patients on Healthy Lifestyles…							
Medical Knowledge	0.10	1.10	-0.89	0.69	0.29	9	0.39
Nutrition Knowledge	0.10	0.74	-0.63	0.43	0.43	9	0.34
Culinary Knowledge	0.20	1.23	-0.68	1.08	-0.51	9	0.31
Prepared to Counsel Patients	-0.10	1.29	-1.02	0.82	0.25	9	0.41
Motivated to Counsel Patients	-0.40	1.17	-1.24	0.44	1.08	9	0.16
Excited to Counsel Patients	-0.50	0.97	-1.20	0.20	1.63	9	0.07

Note: *significant one-tailed *p*<0.05; N = Number. M = Mean. SD = Standard Deviation. CI = Confidence Interval. df = Degrees of Freedom

Counseling-focused survey questions examined participants’ perceived abilities surrounding medical, nutrition, and culinary knowledge as well as their preparedness, motivation, and excitement to counsel patients on healthy lifestyles. From each of the 3 timeframe measurements, improvements in mean scores were reported in participants’ counseling abilities in medical, nutritional and culinary knowledge areas as well as in their preparedness in counseling patients (Table 3). Motivation (*M* = 9.14, *SD* = 1.10) and excitement (*M =* 9.36, *SD* = 0.93) to counsel patients on healthy lifestyles averages were both high (e.g. scores above 9 out of 10) at baseline compared to other counseling areas. To further evaluate counseling characteristics of the participants, Table 4 highlights the statistically significant paired score differences from pre- to post-test time frames which were found in medical knowledge counseling (2.00 [95% CI, 0.47, 3.53]; *p* = 0.01), nutritional knowledge counseling (3.36 [95% CI, 1.50, 5.22]; *p* = 0.00) and culinary knowledge counseling (3.18 [95% CI, 1.10, 5.26]; *p* = 0.00) as well as in preparedness in counseling patients (1.82 [95% CI, 0.70, 2.94]; *p* = 0.00). Post-test to 2-month follow-up data analysis found no statistically significant differences in mean scores in any area of counseling (Table 6).

Culinary knowledge and skills as well as nutrition knowledge saw mean score improvement from baseline to the post-test time; however, both areas declined from the post-test to the 2-month data collection point (Table 3). Nutrition knowledge mean scores were lower at baseline (*M* = 7.00, *SD* = 1.04) compared to culinary knowledge (*M* = 8.07, *SD* = 0.78). The average culinary knowledge and skills scores suggest that participants had elevated culinary proficiency before the study as evidenced by a mean baseline score of 8.07 (*SD* = 0.78) out of 10.

A paired sample *t*-test analysis examined culinary and nutrition knowledge mean scores that changed from pre- to post-test, pre- to 2-month follow-up, and post- to 2-month follow-up periods. Originally the *t*-test analysis was performed with an alpha level of 0.05 and a statistically significant finding (0.71 [95% CI -0.06, 1.47]; *p* = 0.03) was identified for pre- to post-culinary knowledge scores; however; the finding had a confidence interval (CI) which crossed the zero point thus raising concerns about the data significance. Therefore, a re-analysis of the culinary and nutrition knowledge scores was performed and reported at an alpha level of 0.10 and CI of 90% (Table 7). Results of the analysis of culinary knowledge from pre- to post-test using a 90% CI found a statistically significant interaction (0.71 [90% CI 0.08, 1.33]; *p* = 0.03), but neither the pre- to 2-month follow-up (0.35 [90% CI -0.15, 0.84]; *p* = 0.12) nor the post- to 2-month follow-up change scores (-0.23 [90% CI -0.82, 0.37]; *p* = 0.75) were significant.

**Table 7:** Paired Sample T-test Results Comparing Pre-Test, Post-Test and 2-Month Follow-Up Change Scores on Culinary and Nutrition Knowledge

		Mean		90% CI				Significance
Characteristic	N	*Difference*	*SD*	Lower	Upper	*t*	df	(one-sided *p*)
**Culinary Knowledge**								
Pre- to Post-Test	11	0.71	1.14	0.08	1.33	2.05	10	0.03*
Pre- to 2-month Follow-Up	13	0.35	1.00	-0.15	0.84	1.25	12	0.12
Post- to 2-month Follow-Up	10	-0.23	1.03	-0.82	0.37	-0.69	9	0.75
**Nutrition Knowledge**								
Pre- to Post-Test	11	0.46	1.86	-0.06	1.47	0.81	10	0.22
Pre- to 2-month Follow-Up	13	0.15	1.44	-0.57	0.88	0.38	12	0.36
Post- to 2-month Follow-Up	10	-0.30	2.16	-1.55	0.95	-0.44	9	0.66

Note: *significant one-tailed *p*<0.10; N = Number; SD = Standard Deviation; CI = Confidence Interval; df = Degrees of Freedom

Furthermore, there were no statistically significant differences in the analyses of nutrition knowledge for pre- to post-test (0.46 [90% CI -0.06, 1.47]; *p* = 0.22), pre- to 2-month follow-up (0.15 [90% CI -0.57, 0.88]; *p* = 0.36), or post- to 2-month follow-up (-0.30 [90% CI -1.55, 0.95]; *p* = 0.66).

Eating behaviors data analysis indicated that on average participants reportedly ate lunch (*M* = 5.57, *SD* = 2.34) and dinner (*M* = 5.50, *SD* = 2.31) at home more often than breakfast (*M* = 5.29, *SD* = 2.92) at the study baseline (Table 3). After participating in the culinary classes (aka post-test), participants reported on average that they ate breakfast (*M* = 6.55, *SD* = 2.95) and lunch (*M* = 5.91, *SD* = 2.77) at home compared to fewer dinners (*M* = 4.82, *SD* = 2.14). At the 2-month follow-up timeframe, the average number of times breakfast was eaten at home during the week (*M* = 6.15, *SD* = 2.73) had the largest mean increase in frequency from baseline (Table 3). Participants reported that on average they ate pre-prepared meals (e.g. supermarket deli, frozen meals, etc.) on a few occasions before the study (*M* = 2.29, *SD* = 2.37) and even less at the 2-month follow-up (*M* = 2.15, *SD* = 2.85) (Table 3).

Eating meals in restaurants was an infrequent dining experience with fewer than 4 times per week on average at baseline, post-intervention, and at the 2-month follow-up time point (Table 3). Leftovers were consumed at their highest frequency at baseline at approximately 6 times per week on average. Comparing pre- and post-test behaviors, the only statistically significant difference found was a decrease in participants eating leftovers from meals made at home each week (-1.55 [95% CI, -3.09, 0.00]; *p* = 0.03) (Table 4). No statistically significant changes in eating behaviors were identified from pre- to 2-month (Table 5) or post- to 2-month (Table 6) periods.

Lastly, when participants reported their baseline understanding of the scope of practice of a registered dietitian (RD), responses ranged on a five-point Likert scale from strongly disagree to strongly agree. By the end of the study, all participants either agreed or strongly agreed that they understood the role of an RD. Both the pre- to post-test (1.27 [95% CI, 0.67, 1.88]; *p* < 0.001) and pre- to 2-month follow-up (1.08 [95% CI, 0.56, 1.60]; *p* < 0.001) paired samples demonstrated statistically significant improvements in participants understanding an RDs scope of practice. Post-test to 2-month follow-up paired sample *t*-test analysis did not indicate a statistically significant change in scores (*p* = 0.17).

## Discussion

In preparing health professional students to work with clients on achieving nutrition goals, this study focused on 3 main objectives. For the first objective, study participants were found to have statistically significant improvements in their attitudes towards practicing a healthy lifestyle from pre- to post-test assessment periods. Each of the attitude-focused areas surveyed increased, which was the strongest finding of this study. Findings were similar to results on attitudes previously reported by Wood and colleagues, which also indicated improvements in attitudes from baseline responses [14]. Additionally, participants in this study had similar pre-test, post-test, and 2-month follow-up attitudes towards their motivation with a pre-test mean score of 7.64 at baseline and higher at the 2-month follow-up period which is comparable to previous research findings [14]. These results underscore the fact that medical and inter-professional healthcare students have high levels of motivation about practicing a healthy lifestyle. Moreover, study findings suggest that participants not only had elevated levels of motivation but also retained motivation throughout the 2-month follow-up, displaying a lasting impact of motivation. Having a positive attitude as reported from this study and high levels of motivation are factors emphasized within the Social Cognitive Theory and help support behavior change as well as the ability to achieve positive health outcomes [31, 36, 37].

Secondly, participants reported improvements in many of their abilities for counseling clients toward practicing a healthy lifestyle. These findings are similar to research where students were more likely to feel confident discussing nutrition with patients upon completing a culinary experience [13, 33, 38] These study results mirror those previously published which show that students report having the medical knowledge to counsel clients on healthy lifestyles at higher levels than their nutritional or culinary abilities [14] Nutrition and culinary courses are needed to improve the abilities of health professional students in counseling clients on healthy lifestyles. Additionally, results from Wood et al [14] and this study both indicate that students are highly motivated and excited to counsel patients on healthy lifestyles, with mean survey scores of 8.0 out of 10 from Wood et al. and 9.0 out of 10 from this study.

The third study objective focused on identifying any improvements in culinary knowledge skills and nutrition knowledge scores. Interestingly, study participants reported elevated levels of culinary skills with scores over 8 out of 10 at each of the three study timeframes. A statistically significant increase in pre- to post-culinary scores (0.71 [90% CI 0.08, 1.33]; *p* = 0.03) was shown, but the changes that were observed immediately after the intervention (post-test) did not last to the 2-month follow-up time. Nutrition knowledge mean survey scores increased slightly during the study; however, the paired *t*-test analysis indicated no statistically significant difference in scores. Improvements in nutrition knowledge scores were sustained above baseline and even at the 2-month follow-up. Statistically significant improvements in nutrition knowledge could perhaps be gained with the addition of educational sessions or a more focused nutrition curriculum based on formative data collection before any educational sessions.

In the future, culinary programs could consider blending culinary aspects with clinically-focused and knowledge-based activities as those have been well received previously by IPE students [30] Moreover, expanding to additional class sessions beyond the 4 this study offered, or even online didactic nutrition components, could be considered for future studies. These pedagogical strategies may allow for targeted skill development as well as potentially increase the number of students and programs offered [12]. Lastly, study findings imply there was a statistically significant increase in participant understanding surrounding a registered dietitian’s scope of practice. This finding echoes previous research highlighting the important interactions offered through hands-on culinary programs with increasing the understanding of the dietitian’s role in health care and opening the door to future enhanced collaborations [13, 28].

Limitations of the study included a small sample size with an average of 9 students attending the weekly classes and also the possibility of selection bias as only those interested in the study topic would have been more apt to participate. It is unknown why participants choose not to participate in the culinary series, but possibilities could be attributed to factors such as lack of time, inconvenience of the session time slots, peer influence, health and/or safety factors, or many other reasons. Also, while the study questionnaire did achieve 100% face validity from the subject matter experts, it did not meet the content validity ratio (CVR) threshold level required [35] Additional testing and refining of the questionnaire is warranted. Lastly, participant data was self-reported which has inherent limitations in validating.

## Conclusions

Overall, future healthcare providers attending the pilot study indicated that they learned some culinary skills and acquired more nutrition knowledge while being shown that healthy eating is possible on a budget. Research demonstrates that a benefit of culinary programs is that they can decrease the belief that healthy eating is expensive which reinforces the basis of this study [13] Nutrition and culinary programs can also be successful in meeting accreditation standards and competencies which benefit both the students and educational programs [38].

In this study, future dietitians learned to design and implement an educational cooking demonstration including the creation and testing of recipes and lesson plans that meet various dietetic program competencies. The culinary pilot also offered an enhanced opportunity for faculty and student interaction which helped to develop trust beyond a student’s specific discipline. A strong benefit of the pilot study was that all students either agreed or strongly agreed that they understood the role of the registered dietitian by the end of the series. While this study did not analyze any particular dietary changes of participants, it has been previously reported as a benefit of a culinary program whereas fruits, vegetables, and whole grain consumption increased [33].

For future studies, there are several areas of improvement that the researchers suggest. First, additional allied health professional students (e.g. exercise science, perfusion, etc.) should be added to the cohort for a greater variety of diversity and student perspectives. Second, educational objectives, both culinary and nutrition-related, should be paired down, and more focused as session time constraints can become a factor. Third, the survey validity should be addressed with repeated testing of the questionnaire to achieve acceptable content validity levels. Fourth, for greater generalizability of the findings, the authors recommended the intervention be repeated with additional numbers of students to compare outcomes to this pilot study. Finally, the selected recipes for the project should have an ongoing cost evaluation since inflation of grocery prices may impact the feasibility surrounding economical food options. Recipe selection should also be evaluated to maintain the integral connection between the educational outcomes and skill development since many participants already had higher levels of culinary skills coming into the cooking series.

## Electronic supplementary material

Below is the link to the electronic supplementary material.

Supplementary Material 1

## Data Availability

The datasets generated and/or analyzed for the study are not publicly available due to the homogeneous nature of the sample and to protect privacy of the participants but are available from the corresponding author upon reasonable request.

## Abbreviations

CI: Confidence Interval
CVR: Content Validity Ratio
IPE: Interprofessional Education
IRB: Institutional Review Board
M: Mean
SCT: Social Cognitive Theory
SNAP: Supplemental Nutrition Assistance Program
SD: Standard Deviation
USDA: United States Department of Agriculture
