# Supplementary material for: Increasing nutrition knowledge and culinary skills in interprofessional healthcare students: an active learning pilot study
Source: BMC Med Educ. 2025 May 26;25:777. doi: 10.1186/s12909-025-07247-y (PMC12107952; doi:10.1186/s12909-025-07247-y)
Supplement: Supplementary file 1 — Supplementary Material 1 [file 12909_2025_7247_MOESM1_ESM.docx]

**Increasing Nutrition Knowledge and Culinary Skills in Interprofessional Healthcare Students: An Active Learning Pilot Study**

**Questionnaire**

**Directions:** *Please answer the questions below.*

**Email:**

**Age:** *(in years)*

**Gender:**

Male

Female

Prefer Not to Say

**What is your ethnic background?**

White/Caucasian

Asian/Indian

Hispanic

African American

Native American

Other _________

Prefer Not to Say

**Are you married?**

Yes

No

Prefer Not to Say

**Directions**: *For the following questions, please select the best answer for the following topics - Attitudes, Behavior, Counseling Patients on Healthy Lifestyles, Culinary Skills and Knowledge, and Nutrition.*

**ATTITUDES**

Please rate your agreement with each of the following statements from *0-10*, where

*0* = “do not agree at all” to “*10* = “completely agree”

1. I have the **medical** **knowledge** necessary to practice a healthy lifestyle.

0 1 2 3 4 5 6 7 8 9 10

2. I have the **nutritional** **knowledge** necessary to practice a healthy lifestyle.

0 1 2 3 4 5 6 7 8 9 10

3. I have the **culinary theory/knowledge** necessary to practice a healthy lifestyle.

0 1 2 3 4 5 6 7 8 9 10

4. I have the **culinary technique/skills** necessary to practice a healthy lifestyle.

0 1 2 3 4 5 6 7 8 9 10

5. I have the **motivation** necessary to practice a healthy lifestyle.

0 1 2 3 4 5 6 7 8 9 10

6. I can use culinary knowledge and skills to positively impact my **health**.

0 1 2 3 4 5 6 7 8 9 10

**BEHAVIOR**

Please estimate the ***number of times per week*** you engage in the following behaviors.

1. I eat meals from a **restaurant** (e.g. dine-in, delivery, and takeout).

0 1 2 3 4 5 6 7 8 9 10+

2. I eat **pre-prepared meals** NOT from a restaurant (e.g. supermarket deli, frozen,

or microwavable meals, etc.).

0 1 2 3 4 5 6 7 8 9 10+

3. I make or assemble my **breakfast** at home.

0 1 2 3 4 5 6 7 8 9 10+

4. I make or assemble my **lunch** at home.

0 1 2 3 4 5 6 7 8 9 10+

5. I make or assemble my **dinner** at home.

0 1 2 3 4 5 6 7 8 9 10+

6. I eat **leftovers** previously prepared from meals I made or assembled at home.

0 1 2 3 4 5 6 7 8 9 10+

7. For whatever reason, I choose to eat a pre-prepared meal (e.g. either packaged or

from a restaurant) **despite** preferring to eat something I make or assemble at home.

0 1 2 3 4 5 6 7 8 9 10+

**COUNSELING PATIENTS ON HEALTHY LIFESTYLES**

Please rate your agreement with each of the following statements from *0-10*, where

*0* = “do not agree at all” to *10* = “completely agree”

1. I have the **medical** **knowledge** necessary to effectively counsel patients on how

to practice a healthy lifestyle.

0 1 2 3 4 5 6 7 8 9 10

2. I have the **nutritional** **knowledge** necessary to effectively counsel patients on

how to practice a healthy lifestyle.

0 1 2 3 4 5 6 7 8 9 10

3. I have the **culinary** **knowledge** necessary to effectively counsel patients on how

to practice a healthy lifestyle.

0 1 2 3 4 5 6 7 8 9 10

4. I am **prepared** to effectively counsel patients on how to practice a healthy

lifestyle.

0 1 2 3 4 5 6 7 8 9 10

5. I am **motivated** to effectively counsel patients on how to practice a healthy

lifestyle.

0 1 2 3 4 5 6 7 8 9 10

6. I am **excited** to effectively counsel patients on how to practice a healthy lifestyle.

0 1 2 3 4 5 6 7 8 9 10

**CULINARY KNOWLEDGE & SKILLS**

1. The _____ knife technique is where the guiding hand’s fingertips are tucked under

slightly so that the knife rests directly against the knuckles.

1. **claw**
2. classic
3. French
4. Pairing

2. The purpose(s) of ___________ foods is/are to release their flavors, make them easier to cook and easier to eat.

1. dicing
2. chopping
3. mincing
4. **All of the above**

3. Prolonged storage of vegetables may decrease their _____.

a. Price

b. Residue

**c. Crunch or crispness**

d. Odor

4. One teaspoon (tsp) of dried herbs is typically equivalent to what amount of fresh herbs?

1. ½ teaspoon (tsp)
2. ¾ teaspoon (tsp)
3. **1 tablespoon (tbsp)**
4. 2 tablespoons (tbsp)

5. How do you accurately measure oil, milk or water for a recipe?

1. Spoon the liquid into a measuring cup until it’s full. See what the recipe calls for and then very carefully pour out that amount.
2. **Set the liquid measuring cup on a level surface and pour the liquid in, get eye level with the measuring cup, then add in or pour some out until it is at the desired measurement.**
3. While holding the measuring cup up to a light, see if the liquid is close to the amount you need.
4. Add more liquid into the measuring cup than you really need, then pour out however much liquid the recipe calls for.

6. Using the same utensil for preparing raw meat and raw vegetables is likely to cause _____.

- 1. **Cross-contamination which could cause a food-borne illness**
  2. Wilted vegetables which are difficult to prepare
  3. A foul odor on the vegetables making them taste bad
  4. The utensils to become slippery making the food hard to cut

7. Which foods are best for roasting?

1. Collard greens, spinach, peas
2. Beans or Legumes
3. Brisket and boneless chicken breast
4. **Potatoes, butternut squash, parsnips**

8. Match the knives pictured with their function? (knives *maybe be matched more than once)*

Peeling Potatoes ______ **(C)**

Cutting Bread _________ **(A)**

Chopping Celery ______ **(B)**

Slicing Strawberries _____ **(C)**


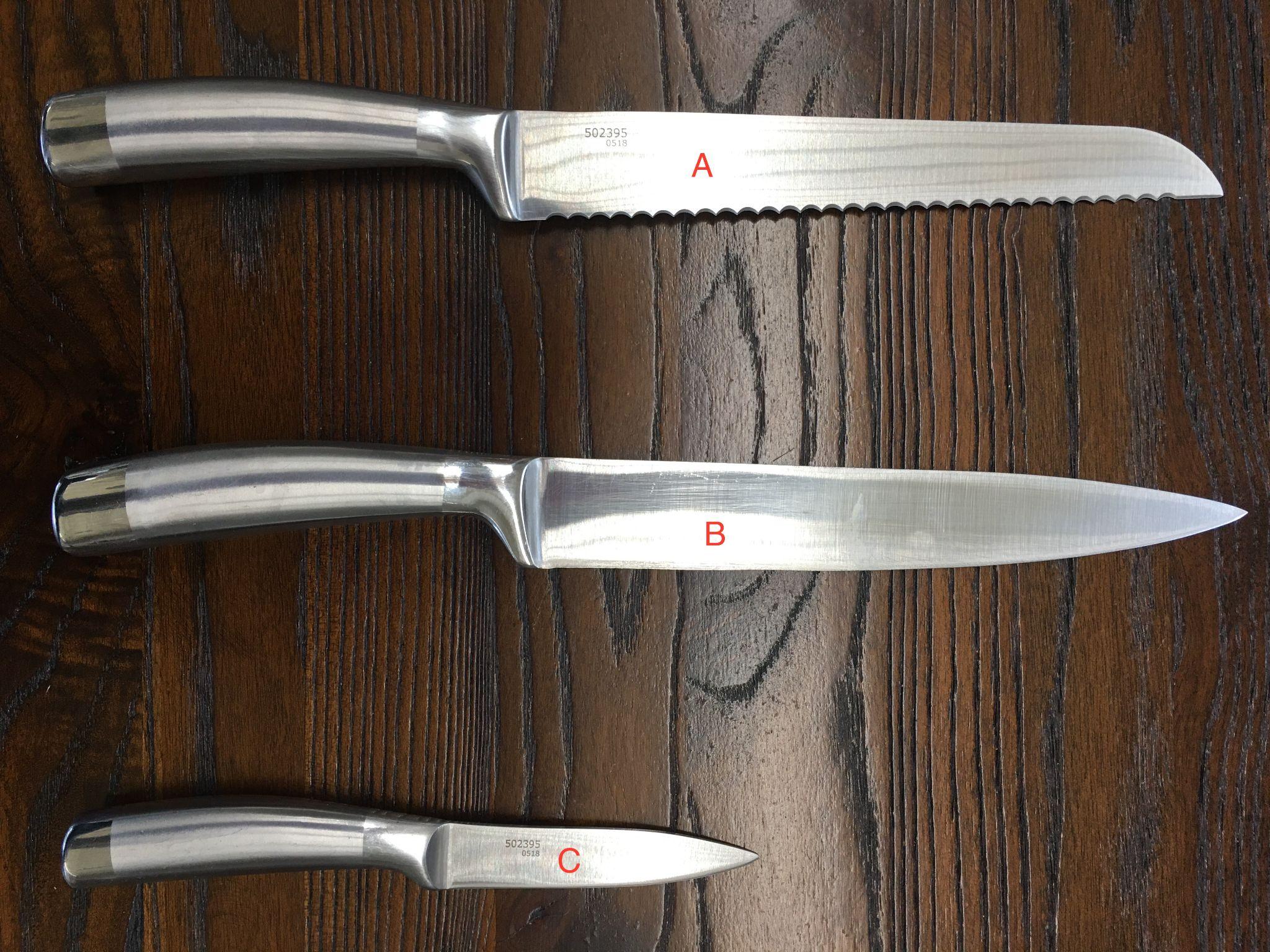


9. To cook foods in a small amount of fat is to _____ them.

1. Steam
2. Bake
3. **Sauté**
4. Fry

10. Which preparation methods preserve the most nutrients for fruits and vegetables?

1. **Steam, Sauté’, Microwave**
2. Fry, Sauté’, Boil, Roast
3. Baked, Fry, Braise
4. Stir-fry, Boil, Dried, Brown

**NUTRITION KNOWLEDGE**

1. Which nutrient directly raises blood sugar?

1. **Carbohydrates**
2. Fats

c. Proteins

d. Vitamins

2. Consuming at least half of all grains as whole grains and choosing dark-green, red and orange vegetables are examples of which diet planning principle?

1. Adequacy
2. Balance
3. **Variety**
4. Moderation

3. The Dietary Approaches to Stop Hypertension (DASH) eating plan emphasizes which of the following foods?

1. Fresh Fruits and Vegetables
2. Low-fat Dairy
3. Poultry, Fish & Nuts
4. **All of the above are emphasized in the DASH eating plan**

4. Which type of fat has been shown to increase the risk for cardiovascular disease?

1. Olive oil
2. **Hydrogenated**
3. Canola oil
4. Soybean oil

5. Flaxseed, walnuts and fatty fish are good dietary sources of _____.

1. Iron
2. Vitamin A
3. **Omega-3 fatty acids**
4. Folate

6. The function of fiber includes ________.

1. Fiber is essential for normal gastrointestinal function
2. Fiber provides bulk for "good" intestinal microbes
3. Fiber provides enhanced gastrointestinal motility and aids in the prevention of constipation and diverticular disease by decreasing transit time and pressure in passing stools
4. **All of the above are functions of fiber**

7. The relationship between energy intake and energy expenditure is termed ______.

1. **Energy balance**
2. Energy calorimetry
3. Energy homeostasis
4. Energy thermogenesis

8. What is the difference between a serving size and a portion size?

1. Serving size is the amount of food eaten in a single eating occasion while a portion size is how much you are supposed to eat.
2. There is not really a difference between portion and serving size.
3. **Portion size is the amount of food eaten in a single eating occasion while a serving size is how much you are supposed to eat.**
4. Portion size is how much of a food that you’ve eaten all day while a serving size is what you’ve eaten at a single meal.

9. The *2020-2025 Dietary Guidelines for Americans* recommends that those aged 14 and

older consume less than 2,300 milligrams (mg) of sodium each day as part of a healthy

eating pattern. One teaspoon (tsp) of salt contains how many milligrams of sodium?

1. 2000
2. **2300**
3. 2500
4. 3000

10. Which would be a good snack choice for a diabetic patient?

a. 34 fresh purple grapes

b. 6 breaded chicken nuggets, 3 packets of ketchup, and 8 oz of unsweet tea

c. 2 slices of sourdough toast topped with 2 tablespoons each of butter and sugar free jelly

**d. 8 oz of reduced-fat milk and ½ fresh banana**

11. I understand the scope of practice of a Registered Dietitian.

1. Strongly Disagree
2. Disagree
3. Neutral
4. Agree
5. Strongly Agree
